# FABP7 and HMGCS2 Are Novel Protein Markers for Apocrine Differentiation Categorizing Apocrine Carcinoma of the Breast

**DOI:** 10.1371/journal.pone.0112024

**Published:** 2014-11-12

**Authors:** Pavel Gromov, Jaime A. Espinoza, Maj-Lis Talman, Naoko Honma, Niels Kroman, Vera Timmermans Wielenga, José M. A. Moreira, Irina Gromova

**Affiliations:** 1 Danish Cancer Society Research Center, Genome Integrity Unit, Copenhagen, Denmark; 2 Department of Pathology, Center for Investigation in Translational Oncology (CITO), School of Medicine, Pontificia Universidad Católica de Chile, Santiago, Chile; 3 Department of Pathology, the Centre of Diagnostic Investigations, Copenhagen University Hospital, Copenhagen, Denmark; 4 Research Team for Geriatric Pathology, Tokyo Metropolitan Institute of Gerontology, Tokyo, Japan; 5 Department of Breast Surgery, Copenhagen University Hospital, Copenhagen, Denmark; 6 Section of Molecular Disease Biology and Sino-Danish Breast Cancer Research Centre, Department of Veterinary Disease Biology, Faculty of Health and Medical Sciences, University of Copenhagen, Copenhagen, Denmark; Okayama University, Japan

## Abstract

Apocrine carcinoma of the breast is a distinctive malignancy with unique morphological and molecular features, generally characterized by being negative for estrogen and progesterone receptors, and thus not electable for endocrine therapy. Despite the fact that they are morphologically distinct from other breast lesions, no standard molecular criteria are currently available for their diagnosis. Using gel-based proteomics in combination with mass spectrometry and immunohistochemistry we have identified two novel markers, HMGCS2 and FABP7 that categorize the entire breast apocrine differentiation spectrum from benign metaplasia and cysts to invasive stages. Expression of HMGCS2 and FABP7 is strongly associated with apocrine differentiation; their expression is retained by most invasive apocrine carcinomas (IAC) showing positive immunoreactivity in 100% and 78% of apocrine carcinomas, respectively, as compared to non-apocrine tumors (16.7% and 6.8%). The nuclear localization of FABP7 in tumor cells was shown to be associated with more aggressive stages of apocrine carcinomas. In addition, when added to the panel of apocrine biomarkers previously reported by our group: 15-PGDH, HMGCR and ACSM1, together they provide a signature that may represent a golden molecular standard for defining the apocrine phenotype in the breast. Moreover, we show that combining HMGCS2 to the steroidal profile (HMGCS2+/Androgen Receptor (AR)+/Estrogen Receptor(ER)-/Progesteron Receptor (PR)- identifies IACs with a greater sensitivity (79%) as compared with the steroidal profile (AR+/ER-/PR-) alone (54%). We have also presented a detailed immunohistochemical analysis of breast apocrine lesions with a panel of antibodies against proteins which correspond to 10 genes selected from published transcriptomic signatures that currently characterize molecular apocrine subtype and shown that except for melanophilin that is overexpressed in benign apocrine lesions, these proteins were not specific for morphological apocrine differentiation in breast.

## Introduction

Apocrine carcinoma of the breast exhibits the same histological growth pattern as invasive ductal carcinoma of no special type, and is currently diagnosed on basis of the presence of characteristic apocrine-type epithelial cell morphology observed in more than 90% of tumor cell mass. These tumors represent a relatively rare subtype, constituting less than 5% of all breast cancers [1], [2]. Recently, Dellapasqua and coauthors reported a frequency of apocrine carcinoma of 0.8% after analyzing a cohort of 6971 breast cancer patients [3]. This high discrepancy is most likely because there is no consensus on standardized reproducible diagnostic criteria as the current WHO classification of breast malignancies provides an imprecise definition of apocrine carcinoma of the breast [4], a fact that has produced controversial and heterogeneous conclusions in the scientific literature in terms of a precise immunohistochemical profile and molecular classification of invasive apocrine carcinomas (IACs) [1], [5], [6], [7], [8], [9]. Moreover, apocrine differentiation is detected in several other breast tumor subtypes including papillary, micropapillary, tubular, and lobular carcinoma [9]. In addition to characteristic morphological features IACs are generally accepted to have a distinct hormonal profile, being estrogen receptor (ER) and progesterone receptor (PR) negative, but androgen receptor (AR) positive [10]. Again, it should be noted that throughout the years IACs have been reported as ER positive in 3.8–60% of cases, PR positive in 4.8%–40% and AR positive in 56%–100% [1], underscoring the variability in observation reported for these tumors. There are not much data regarding the clinical outcome of those tumors and the results are not compelling enough partly because of limited numbers of samples selected for the analysis [9]. A comprehensive study published recently has revealed a significantly worse disease free survival for pure IACs as compared with invasive ductal carcinoma (IDC) [3].

A few years ago several transcriptomic studies were performed with the aim to classify these types of breast malignancy at the molecular level. In the gene profiling study carried out by Perou and coauthors, IACs clustered within the basal-like subtype of breast carcinomas [11]. Thereafter, Farmer and colleagues [12] identified a subset of breast tumors characterized by increased androgen signaling and a distinctive expression profile, which they called “molecular apocrine” as these lesions did not exhibit all the histopathological traits that are characteristic of classical apocrine carcinomas. Molecularly defined apocrine carcinomas include tumors that share some common expression characteristics with the HER2+ group (ER-/PR-/HER2+) in the Stanford classification as well as with some lesions that exhibit features of the basal-like/triple negative group (high grade lesions; ER-/PR-/HER2-). It was also shown that it is possible with microarray data to divide mammary tumors into 3 major groups based on steroid hormone status: luminal (ER+/AR+), basal (ER−/AR−), and molecular apocrine (ER−/AR+) with a certain association between apocrine histology and molecular apocrine type [13]. Finally, a meta-analysis study performed by Sanga and colleagues [14] showed that the subgroups described by Farmer and Doane are highly similar and both predict the molecular apocrine subset in other cohorts. To date, however, the relationship between molecular apocrine breast carcinoma and histopathologically defined apocrine tumors remains questionable [15].

With the aim to develop molecular criteria to reproducibly categorize IACs at the protein level we have undertaken a systematic proteomic analysis of well-defined set of apocrine carcinomas aimed at identifying biomarkers that may characterize and subtype these lesions to a greater detail, and to search for targets that may lead to the development of novel targeted therapies and chemoprevention strategies [2], [16], [17], [18], [19]. Accordingly, we have identified a number of apocrine protein markers that include 15-prostaglandin dehydrogenase (15-PGDH) and acyl-CoA synthetase medium-chain family member 1 (ACSM1) which together with a set of categorizing markers that are predominantly expressed (AR, CD24) or not expressed (bcl-2, GATA-3) by apocrine metaplastic lesions in benign breast lesions, were proven to be specific for both apocrine ductal carcinoma *in situ* (ADCIS) and IAC [2], [16], [17], [19], [20]. This apocrine signature has been shown to identify unambiguously 13 out of 14 ADCIS (92.9%) and 20 out of 33 (60.6%) IACs in a well characterized set of apocrine carcinomas [2] in which more than 90% of the tumor cells exhibited cytological features typical of apocrine cells [21]. Here we describe two additional markers, brain fatty acid binding protein (FABP7) and hydroxymethylglutaryl (HMG)-CoA synthase 2 (HMGCS2), which in combination with markers in the protein signature described previously [19] allowed to identify ADCISs and IACs that failed to be detected in previous studies. Moreover, our results demonstrate that HMGCS2 added to the steroid hormone receptor signature (ER-/PR-/AR+) identifies apocrine tumors from other breast cancer subtypes with greater sensitivity as compared to steroid receptor profile alone. We have also presented a detailed immunohistochemistry (IHC) analysis of a set of proteins corresponding to 10 genes selected from transcriptomic signatures that currently characterize molecular apocrine subtype [12], [13], [14], [22] to evaluate the complementarity of these two approaches.

## Results

### Determination of the specificity of FABP7 and HMGCS2 antibodies

The sensitivity and specificity of antibodies are critical parameters in the design and development of reliable IHC-based assays for analysis and validation of potential biomarkers. To determine if FABP7 and HMGCS2 antibodies are specific enough in terms of IHC staining to identify benign and malignant apocrine lesions [2], [16] we used a three-pronged strategy developed in our laboratory and described in detail elsewhere [23]: (i) analysis of potential cross-reactivity by 2D Western blotting (2D-WB), (ii) mass spectrometry validation of corresponding silver-stained protein spots superimposed with 2D-WB and (iii) IHC experiments with blocking of antibodies with a corresponding protein/epitope. This approach combines the intrinsic sensitivity and cellular resolution of IHC, with the specificity of 2D-WB and MS-based identification, and the overall protein analysis capability of gel-based proteomics [24].

**Figure 1:**
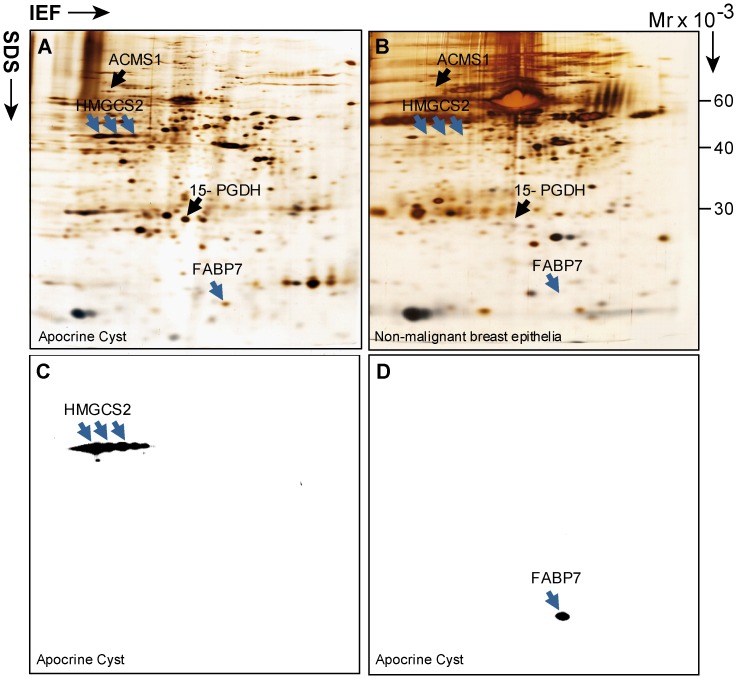
2D PAGE and 2D Western blot analysis of normal breast lesions and apocrine cyst. (A) 2D silver stained gel of protein lysate from apocrine microcyst excised from tumor biopsy of patient 95 (GrI; ER^+^/PR^+^/AR^+^/HER2^1+^). Positions of FABP7 and HMGCS2 identified by mass spectrometry (MS) are indicated by blue arrows. Positions of apocrine differentiation, markers, 15-PGDH and ACSM1, described in our previous studies are indicated by black arrows for reference. (B) 2D silver stained gel of protein lysate from distant normal (app. 3–4 cm from tumor mass) breast lesion resected from mastectomy of patient 121 (GrII; ER^+^/PR^+^/AR^+^/HER2^2+^). HMGCS2 and FABP7 have been identified by MS and are indicated by blue arrows. Positions of HMGCS2, FABP7 (blue arrow) and 15-PGDH, and ACSM1 (black arrows) are indicated. The positions of HMGCS2 and FABP7 on the 2D gel image (B) are located by matching of corresponding gel images by PDQUEST software. (C and D) 2D Western blot of protein lysate from the same apocrine microcyst as in (A) developed either with anti HMGCS2 or anti FABP7 antibodies.

In previous studies we demonstrated overexpression of FABP7 and HMGCS2 by apocrine cells as compared to their normal breast epithelial cell counterparts [2]. 2D-PAGE/MS/2D-WB analyses were performed on tissue lysates obtained from six patients and the results for one matched apocrine cyst/normal epithelia pair are illustrated in Figure 1. As shown, the anti-HMGCS2 antibody detected several train-line protein spots of similar Mw (about 57 kDa) (Figure 1A and C) compatible with multiple post-translational modifications of the protein [25], [26]. The identity of these spots as HMGCS2 was confirmed by MS analysis (data not shown). The antibody recognizing FABP7 detected a single spot with Mw ≅15 kDa (Figure 1A and D). No protein spots matched to the positions of FABP7 and HMGCS2 were detected on the 2D gel of normal breast tissue (Figure 1B). The results show that HMGCS2 and FABP7 are recognized by their corresponding antibodies, and, most importantly, cross-reactivity was detected for neither HMGCS2 nor FABP7 with any of the thousands of proteins resolved by 2D PAGE, demonstrating a high specificity for these antibodies. In addition, to exclude the possibility that anti-FABP7 may react with some close homologues from the FABP protein family [27], we performed 2D–WB with protein extracts from several triple-negative breast cancer samples in which we had previously observed aberrant expression of three other FABP protein family members, namely, FABP3, FABP4 and FABP5 [23]. As seen in Figure S1, anti-FABP7 specifically recognized only a single spot corresponding to FABP7 and no cross reactivity was detected with FABP3, FABP4 or FABP5. The identities of all protein spots were determined by mass spectrometry analysis (data not shown). The high specificity of both antibodies was further confirmed by IHC experiments with blocking of antibodies with a corresponding full length recombinant protein (data not shown). Based on these results it can be concluded that the antibodies against FABP7 and HMGCS2 recognize their cognate antigens with high specificity and, thus, can be incorporated into the panel of antibodies used in IHC experiments to distinguish the cell type that express proteins of interest within heterogeneous clinical samples.

### FABP7 and HMGCS2 are highly expressed in benign breast lesions with apocrine differentiation

**Figure 2:**
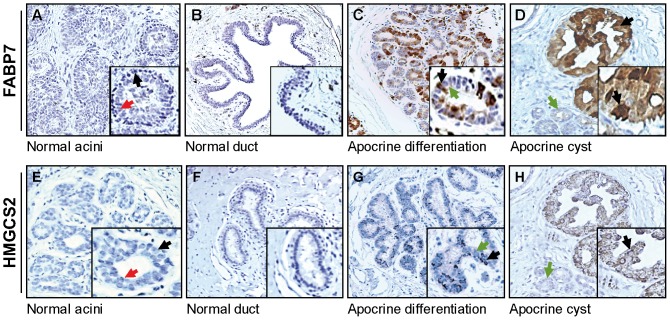
Immunohistochemical analysis of FABP7 and HMGCS2 expression in benign breast lesions with apocrine differentiation. FFPE sections of normal breast and benign breast lesions with apocrine differentiation adjacent to tumor were stained with antibodies against FABP7 (upper panel) and HMGCS2 (low panel). (A) and (E) shows serial sections of normal breast tissue. Luminal and basal/myoepithelial cells are indicated by red and black arrows, respectively. (B) and (F) show sections of large normal ducts. (C) and (G) show serial sections of breast lesions with benign apocrine differentiation (apocrine adenosis). Positive and negative luminal cells are indicated by black and green arrows, respectively. (D) and (H) show serial sections of lesions with apocrine cysts. Apocrine cysts with apical snouts and normal small ducts are indicated with black and green arrows, respectively. Magnification: x10. Representative areas for each staining are shown in higher magnification (x20). The cut-off values for FABP7 and HMGCS2 are specified in Materials and Methods.

**Figure 3:**
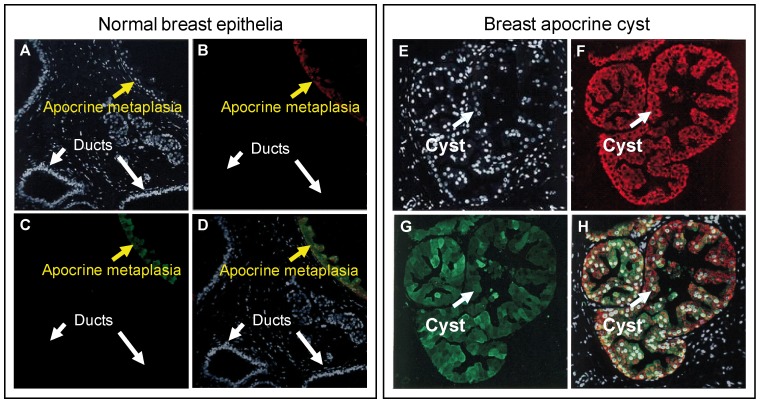
FABP7 and HMGCS2 are colocalized in lesions with apocrine differentiation. Indirect double-label immunofluorescence analysis of normal breast lesion with apocrine metaplasia (left panel) and apocrine cyst sections (right panel) reacted with FABP7 (subpanels B and F) and HMGCS2 (subpanels C and G). Sections were counterstained with the nuclear stain DAPI (blue channel). Merge images are shown on subpanels (D) and (H), respectively.

Having ascertained the strict specificity of the antibodies recognizing FABP7 and HMGCS2, we investigated their expression profiles by IHC on serial sections of lesions with various forms of benign apocrine metaplasia. Thus, we evaluated the percentage of positivity/negativity for HMGCS2 and FABP7 in 2 sets of breast tissue samples, namely, non-malignant breast tissue dissected from the areas adjacent to tumors (28 patients with various types of breast tumors, Table S1) and 13 benign apocrine microcysts obtained after surgery (Table S2). Expression of cytoplasmic FABP7 and HMGCS2 as well as nuclear FABP7 was considered positive whenever we observed more than 60% of cytoplasmic and above 1% of nuclear staining, respectively. Representative IHC patterns are shown in Figure 2. FABP7 nuclear and cytoplasmic staining was not evaluated independently because of limited number of samples presented in each group. As seen, normal terminal ductal lobular units and normal ducts in breast areas adjacent to tumor showed positivity for both proteins neither in luminal cell nor in basal/myoepithelial cells (Figure 2A and B and Figure 2E and F). However, areas with morphological signs of apocrine differentiation exhibited sporadically positive mosaic staining (Figure 2C and G, respectively). Most notably, apocrine cysts exhibited a high immunoreactivity for both HMGCS2 and FABP7 (Figure 2D and H, respectively). To confirm these observations and to justify intracellular localization of both proteins, we examined the expression pattern of HMGCS2 and FABP7 in apocrine cysts by double immunofluorescence (Figure 3). As expected, normal mammary epithelial cells showed little or no evidence of staining with either antibody (Figure 3, left panel). On the contrary, lesions with apocrine metaplasia (Figure 3, left panel) and apocrine cysts (right panel) were highly positive for both proteins. Taken together these experiments showed a preferential expression of HMGCS2 and FABP7 in benign breast lesions undergoing apocrine differentiation. Additionally, non-apocrine flat cysts (type II) that were detected within analyzed lesions were completely negative for both HMGCS2 and FABP7 in all cases (data not shown).

Based on the IHC scoring we have evaluated the percentage of positivity/negativity of both proteins in apocrine cysts as compared to non-apocrine lesions and shown that FABP7 was positive in 12 out of the 13 apocrine cyst samples and negative in 22 out of the 28 non-apocrine samples (92,3% and 78,6%, respectively; p<0.0001). Accordingly 10 out of the 13 apocrine cyst samples (76,9%) and 26 out of 28 non-apocrine ones (92,8%), were classified for HMGCS2 as positive and negative, respectively (p<0.001). These results imply that FABP7 and HMGCS2 can be considered as novel biomarkers for breast apocrine differentiation that together with the apocrine biomarker panel reported by us in previous studies, 15-PGDH+ HMG-CoA reductase+ and ACSM1+ [2], [16], [19], [28] may represent the golden standard for defining the breast apocrine phenotype.

### Expression of FABP7 and HMGCS2 by apocrine carcinoma

**Figure 4:**
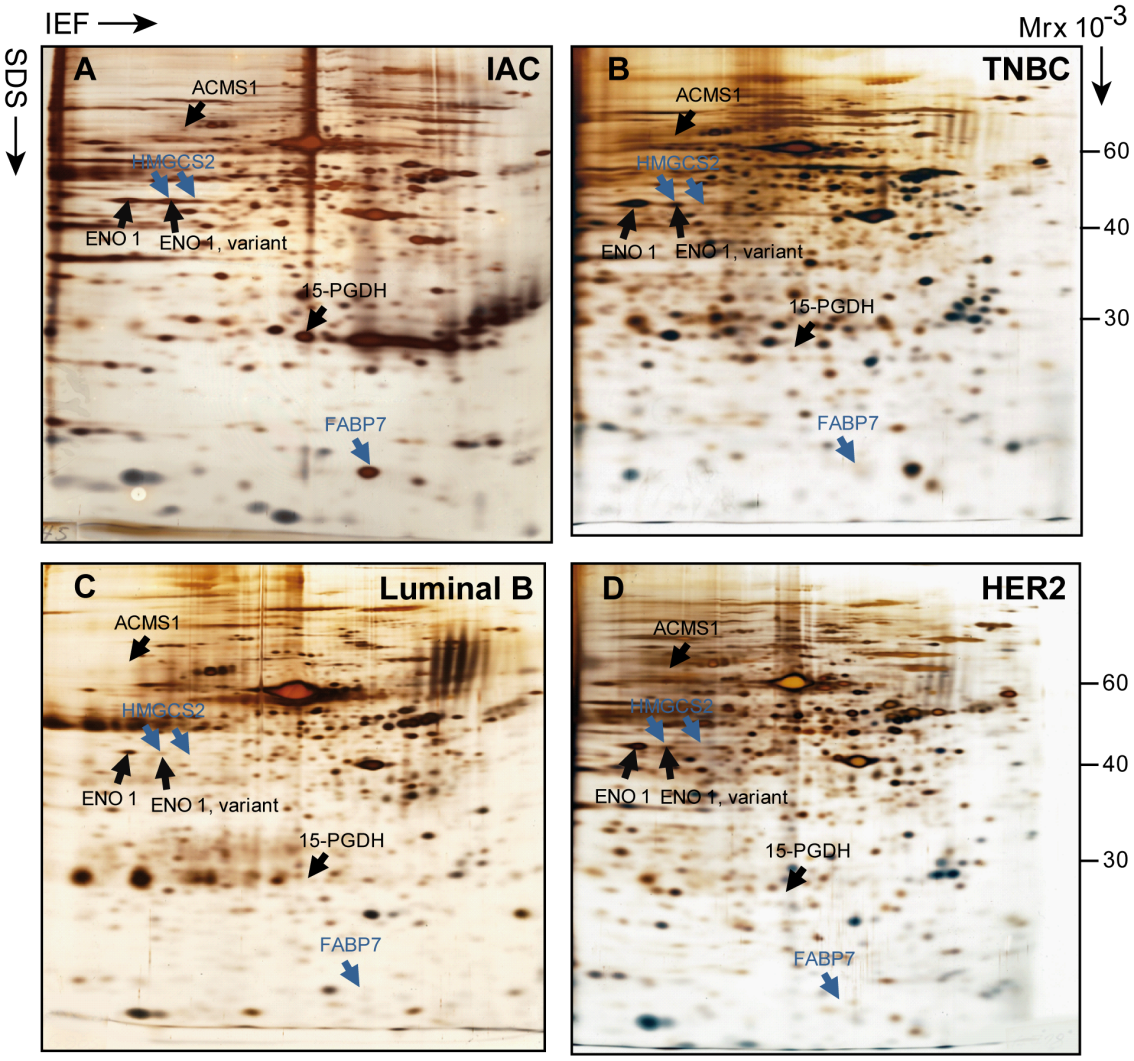
2D PAGE analysis of FABP7 and HMGCS2 expression among breast cancer subtypes. The representative images of IEF 2D PAGE of protein lysates prepared from frozen sections of 4 breast tumor subtypes: IAC (A), TNBC (B), Luminal B (C) and Her2+(D). HMGCS2 and FABP7 have been identified by MS and indicated by blue arrows. The positions of HMGCS2 and FABP7 on the 2D gels of TNBC, Luminal B and HER2 were determined by matching of corresponding images by PDQUEST software. Alpha-enolase variants, identified by MS, are co-migrated with HMGCS2 and indicated by black arrows. Two other IAC markers, 15-PGDH and ACSMS1 described in our previous studies are shown for reference. IAC =  invasive apocrine carcinoma; TNBC =  triple negative breast cancer. Tumors have been stratified as specified in Materials and Methods.

**Figure 5:**
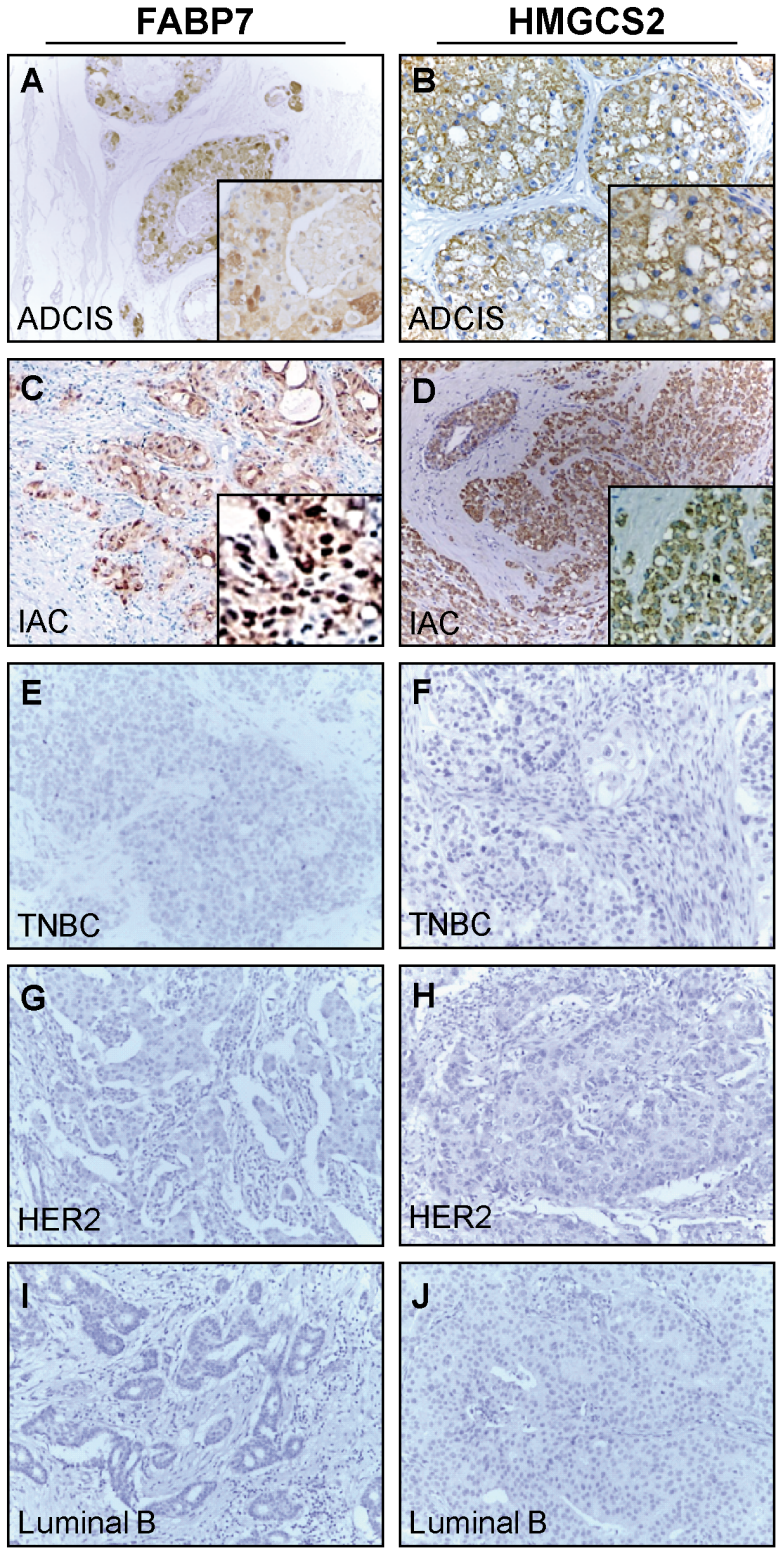
Immunohistochemical expression analysis of FABP7 and HMGCS2 among breast cancer subtypes. The representative IHC images of FFPE sections from 5 breast tumor subtypes immunostained with antibodies against FABP7 (A,C,E,G and I) and HMGCS2 (B,D,F,H and J). ADCIS  =  apocrine ductal carcinoma *in situ*; IAC =  invasive apocrine carcinoma; TNBC =  triple negative breast cancer. Magnification: x10. Representative areas are shown in higher magnification (x20) The cut-off values for FABP7 and HMGCS2 are specified in Material and methods. Tumors have been stratified as specified in Materials and Methods.

The baseline of expression for HMGCS2 and FABP7 in apocrine carcinomas at the protein level was first evaluated in a selected number of well differentiated IACs (6 samples) using 2D PAGE and PDQUEST-based image analysis as compared to TNBC (6 samples), Luminal B (7 samples) and HER2 (7 samples) subtypes of which there was fresh frozen tissue. The representative 2D images of these breast cancer subtypes are shown in Figure 4. All six of the analyzed IACs expressed both proteins (Figure 4A) as it was determined by MS analysis and confirmed by 2D WB (data not shown). No traces of these proteins were found in any of the 3 other breast cancer subtypes analyzed by 2D PAGE (Figure 4B, C and D). Notably, the acidic isoform of HMGCS2 detected in IACs is co-migrated with the basic variants of alpha-enolase (Figure 4A), a protein that is highly expressed in all tumor subtypes analyzed and, as a result, may mask the identification of HMGCS2 by 2D PAGE in these samples. Hence, we performed IHC analysis of these non-apocrine samples together with ADCIS and IAC and confirmed that both proteins are expressed only by apocrine carcinomas as exemplified in Figure 5. As seen, no positive staining for HMGCS2 and FABP7 was observed in TNBC, Luminal B and HER2 samples (Figure 5E–J). It is noteworthy, that in both apocrine carcinoma types, ADCIS and IAC, HMGCS2 displayed only cytoplasmic staining pattern (Figure 5B and D). FABP7 also exhibited cytoplasmic intracellular localization in both apocrine carcinomas, however, in invasive stages, while retaining in cytoplasm, it was also detected in the nuclei with positivity thresholds more than 1% (Figure 5C).

**Figure 6:**
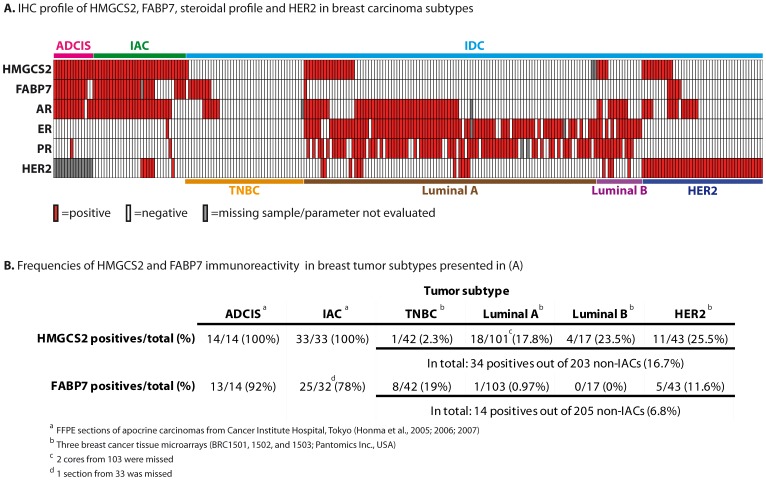
Expression profile of FABP7, HMGCS2 and other markers among breast cancer subtypes. (A) The diagram presents immunohistochemical profiles of ADCIS, IACs and invasive ductal carcinomas (TNBC, Luminal A Luminal B and HER2) reacted with antibodies against HMGCS2, FABP7, AR, ER, PR and HER2. The rows indicate the expression of particular protein in every core in breast cancer TMA (BRC1501, 1502 and 1503; Pantomics INC, USA): red box – positive staining, white box – negative staining, grey box – parameter was not determined. (B) Frequencies of positives for HMGCS2 and FABP7 among breast cancer subtypes. ADCIS =  apocrine ductal carcinoma *in situ*; IAC =  apocrine carcinoma; IDC =  invasive ductal carcinoma; TNBC =  triple negative breast cancer. The cut-off values for HMGCS2 and FABP7 are specified in Materials and Methods. The cut-off values for ER, PR, AR and HER2 are specified in the Table S3 and S4.

Based on these results we proceeded to test the putative two-protein signature within the same unique Japanese collection of apocrine carcinomas [21], [29], [30] that was used to validate the previous protein signature [19]. In this large, well characterized cohort, which is composed of 14 apocrine ductal carcinoma *in situ* (ADCIS) and 33 IACs, more than 90% of the tumor cells exhibited morphological features typical for apocrine cells [19], [29]. All 14 ADCIS (100%) stained positively for HMGCS2 and 13 out of 14 (92%) were immunoreactive for FABP7 (Figure 6, Table S3) with weak intensity of cytoplasmic staining (Table S3). As it was shown previously, all samples did not express ER, PR, Bcl2 and GATA3, but were positive for CD24 and AR (with one exception – ADCIS 12) [19]. The frequencies of HMGCS2 and FABP7 in 33 IACs as evaluated by IHC are shown in Figure 6 and Table S4. HMGCS2 and FABP7 were positive in 100% (33/33) and 78% (25/32) of IACs, respectively. Nuclear localization of FABP7 was associated with invasive stage of apocrine carcinomas as it was detected in all 33 IAC samples but was not observed in any of the 14 ADCISs (Table S3 and S4).

### Expression of FABP7 and HMGCS2 by other breast carcinoma subtypes

To determine whether FABP7 and HMGCS2 are exclusively expressed by apocrine carcinoma we analyzed expression patterns of these putative biomarkers in a set of 205 non-apocrine breast tumors: 42 TNBC (21%), 103 Luminal A (50,2%), 17 Luminal B (8,3%) and 43 HER2+ (20,5%) contained in three commercially available tissue microarrays (BRC1501, 1502, and 1503; Pantomics Inc.) (Figure 6, Table S5). The frequencies of positivity for HMGCS2 and FABP7 as compared to IACs are summarized in Figure 6B. In total, FABP7 positives were detected in 14 out of the 205 breast non-apocrine carcinomas (6,79%): 8 out of the 42 TNBCs (19,04%); 1 out of the 103 Luminal A subtypes (0,97%); in none out of the 17 Luminal B (0%) and in 5 out of the 43 HER2+ (11,62%). HMGCS2 was expressed in 1 out of 42 TNBCs (2,38%); 18 out of 101 Luminal A (17,8%); 4 out of 17 Luminal B (23,5%) and 11 out of 43 HER2+ (25,5%), in total: 34 out of the 203 non-apocrine breast carcinomas (16,7%) (Figure 6B). The Fisher exact test confirmed a statistical significant difference in frequencies of HMGCS2 and FABP7 positives between IACs and Luminal A, Luminal B, HER2+ and TNBC groups (p<0.0001 for both proteins). No association was found between HMGCS2 expression and ER or PR status, however, FABP7 is significantly more positive among ER- and PR- breast cancers (p<0.0001).

Given that androgen receptor (AR) is widely expressed in various breast cancer types [31] and leaning on our findings that HMGCS2+/AR+/ER-/PR- phenotype is highly frequent in both ADCIS and IAC subgroups (Figure 6A, Tables S3 and S4), we calculated the frequency of this four-protein signature in non-apocrine Pantomics dataset and revealed only 7 samples (3,5%) with a combination of HMGCS2+/AR+/ER-/PR- (Table S5, corresponding rows are highlighted by Thick Box Border). This finding implies the contention that the HMGCS2+/AR+/ER-/PR- profile is associated with breast apocrine malignancy diagnosed by morphological criteria (p<0.0001) with a sensitivity of 78,8% and a specificity of 96,6% (using a defined threshold). Thus, HMGCS2+/AR+/ER-/PR- profile exhibited a significantly higher sensitivity as compared with the AR+/ER-/PR- phenotype, which only provides a sensitivity of 54,17% for apocrine tumors. Similar statistical evaluation performed for FABP7 demonstrated that FABP7+/AR+/ER-/PR- phenotype exhibited 75% of sensitivity and 94,7% of specificity, outperforming also the steroidal profile alone.

Interestingly, among 7 non-apocrine samples showing HMGCS2+/AR+/ER-/PR- profile 6 were HER2+ (86%; remaining sample was classified as a borderline) (Table S5), whereas in the entire cohort of 203 samples, HER2 positives were observed in 58 samples (28,6%), indicating that among tumors outside apocrine carcinoma the phenotype HMGCS2+/AR+/ER-/PR- is correlated with HER2 amplification (p<0.003). The high percentage of expression of HMGCS2 by HER2+ tumors was further confirmed by IHC using an additional set of 16 samples of HER2+ lesions collected from the department of pathology, Rigshospitalet, Copenhagen University Hospital (data not shown).

### Expression of apocrine differentiation markers in the MDA-MB-453 cell line model for apocrine breast carcinoma

The MDA-MB-453 breast cancer cell line obtained from a malignant pleural effusion of a 48-year-old female is characterized by AR+/ER-/PR-/HER2- profile is often used as a model to study the molecular processes underlying apocrine breast malignancy [32], [33], [34]. In view of this fact, we examined the expression of FABP7 and HMGCS2 together with several other apocrine protein markers in the MDA-MB-453 breast cancer cell line. IHC analysis of MDA-MB-453 cells has been performed using a battery of antibodies against FABP7, HMGCS2, 15-PHGDH, ACSM1 and GCDFP-15. Our data have shown that, in contrast to ADCIS and IACs, MDA-MB-453 cells do not express either the FABP7 or HMGCS2 (Figure S2). This cell line was also negative for 15-PGDH and ACSM1 as well as for GCDFP-15 (Figure S2), which is often considered as an apocrine marker protein in the breast. The latter result is in agreement with the study by Vranic and co-authors [35], who recently updated the molecular profile of the MDA-MB-453 cell line and concluded that these cells may not be an ideal model of breast apocrine carcinomas. Consequently, our data also support this output suggesting that, although the MDA-MB-453 cells share certain features with apocrine breast carcinoma their metabolic phenotype remains plastic and may not reproduce certain expression patterns and pathways which are characteristic for apocrine differentiation in breast.

### Evaluation of the transcriptomic molecular apocrine signature at the protein level

**Figure 7:**
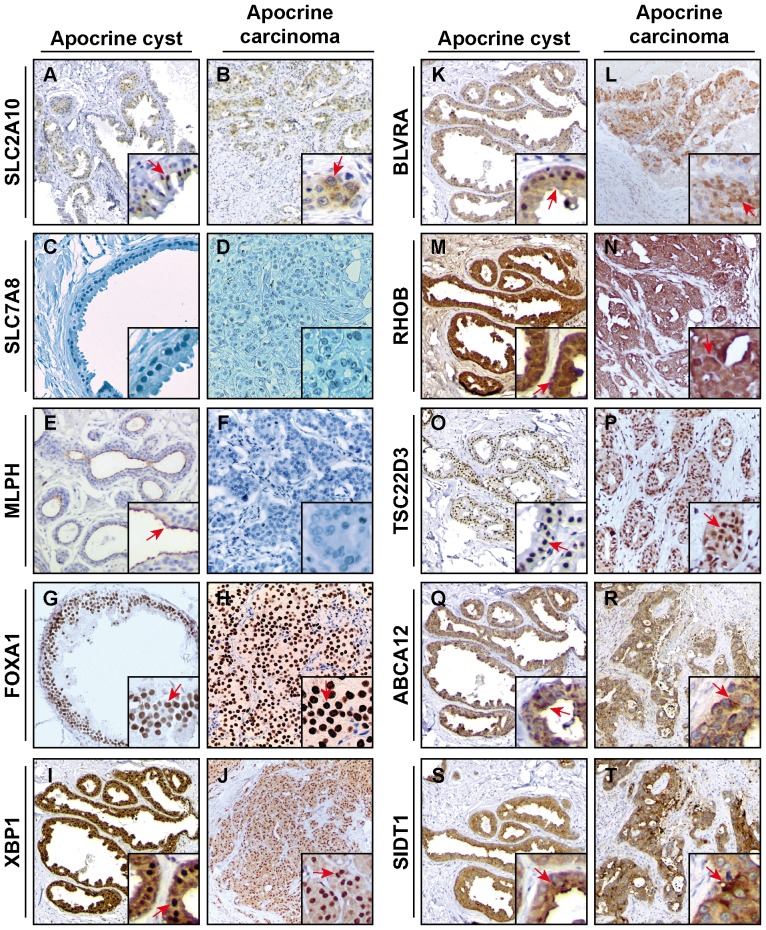
Immunohistochemical analysis of molecular apocrine markers derived from trancriptomics data within the apocrine cyst and carcinoma dataset. Representative staining of FFPE of apocrine cysts and IACs with antibodies against SLC2A10 (Glucose transporter type 10, sections A and B) - cytoplasmic immunoreactivity; SLC7A8 (L-type amino acid transporter 2, sections C and D) - mainly cytoplasmic and membranous immunereactivity; MLPH (Melanophilin, sections E and F) - cytoplasmic and luminal membranous immunoreactivity; FOXA1 (Hepatocyte nuclear factor 3-alpha, sections G and H) – nuclear staining; XBP1 (X-box-binding protein 1, sections I and J) – mainly nuclear and cytoplasmic immunereactivity; BLVRA (Biliverdin reductase A, sections K and L) - cytoplasmic immunoreactivity which combined with rare nuclear positivity; RHOB (Rho-related GTP-binding protein RhoB, sections M and N) - cytoplasmic immunoreactivity; TSC22D3 (TSC22 domain family protein 3, sections O and P) - mainly nuclear and cytoplasmic immunereactivity; ABCA12 (ATP-binding cassette sub-family A member 12, sections Q and R) - cytoplasmic immunoreactivity and SIDT1 (SID1 transmembrane family member 1, sections I and G) - cytoplasmic immunoreactivity. Magnification: x10. Representative areas are shown in higher magnification (x20) and examples of positive staining are indicated by red arrows.

Recently, a gene expression signature called “molecular apocrine” has been proposed based on transcriptomic studies of breast cancer tumors [12], [14]. The molecular apocrine group is AR positive but ER negative, with an increased androgen signaling and some morphological features of apocrine tumors, lacking, however, the strict criteria for diagnosis of classical apocrine carcinomas. To our knowledge, the reliability and significance of the apocrine breast carcinoma molecular signature has not yet been systematically investigated at the protein level. In order to find out to what extend the “molecular apocrine” signature correlates to the expression pattern of corresponding proteins in various breast tumor subtypes we performed a detailed IHC analysis of a 10-protein set selectively chosen from transcriptomic apocrine signature. The selection of the candidate genes was made on the following criteria: (i) their presence in the signatures described by the molecular apocrine meta-analysis as major top candidates [14] and (ii) the availability of specific antibodies to the corresponding proteins suitable for immunohistochemical detection. Following these landmarks, we have chosen 10 genes consistently associated to the “molecular apocrine” signature: ABCA12 (ATP-binding cassette sub-family A member 12), BLVRA (Biliverdin reductase A), FOXA1 (Hepatocyte nuclear factor 3-alpha), MLPH (Melanophilin), RHOB (Rho-related GTP-binding protein RhoB), SIDT1 (SID1 transmembrane family member 1), SLC2A10 (Glucose transporter type 10), SLC7A8 (L-type amino acid transporter 2), TSC22D3 (TSC22 domain family protein 3) and XBP1 (X-box-binding protein 1). The expression pattern of selected candidates was tested by IHC staining on paraffin sections prepared from selected pairs of benign apocrine cyst and IAC, and representative IHC images are shown in Figure 7. Very low immunoreactivity/negativity was observed in both benign and malignant lesions for SCL2A10, SLC7A8 and MPLH (Figure 7A and B; C and D, E and F, respectively), the latter has a distinct apical expression pattern in apocrine cysts. The other seven proteins displayed positive staining of various intensities and localizations in both types of analyzed apocrine lesions: FOXA1, XBP1, BVLRA and RHOB showed positive cytoplasmic and nuclear staining in both cysts and IAC cells (Figure 7G and H, I and J, K and L, M and N, respectively); TSC22D3 was immunoreactive in nuclei of cysts and in nuclei/cytoplasm of IAC cells (Figure 7O and P), whereas ABCA12 and SIDT1 showed only cytoplasmic localization in both types of lesions (Figure 7Q and R, S and T, respectively).

**Figure 8:**
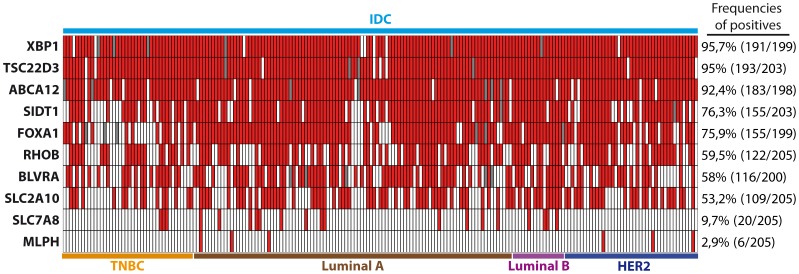
Protein expression profile of transcriptome-derived apocrine markers among breast cancer subtypes. The diagram presents immunohistochemical profiles of four breast tumor subtypes, TNBC, Luminal A Luminal B and HER2, reacted with antibodies against ten transcriptome-derived apocrine markers: XBP1, TSC22D3, ABCA12, SIDT1, FOXA1, RHOB, BLVRA, SLC2A10, SLC7A8 and MLPH. The rows indicate the expression of particular protein in every core in breast cancer TMA (BRC1501, 1502 and 1503; Pantomics INC, USA): red box – positive staining, white box – negative staining grey box – parameter was not determined. Corresponding frequencies of positives are shown on the right side of the diagram. Samples were considered as positive if 10% or more of the cells showed a clear positive staining with the antibodies. IDC =  invasive ductal carcinoma; TNBC =  triple negative breast cancer. Tumors have been stratified as specified in Materials and Methods.

**Figure 9:**
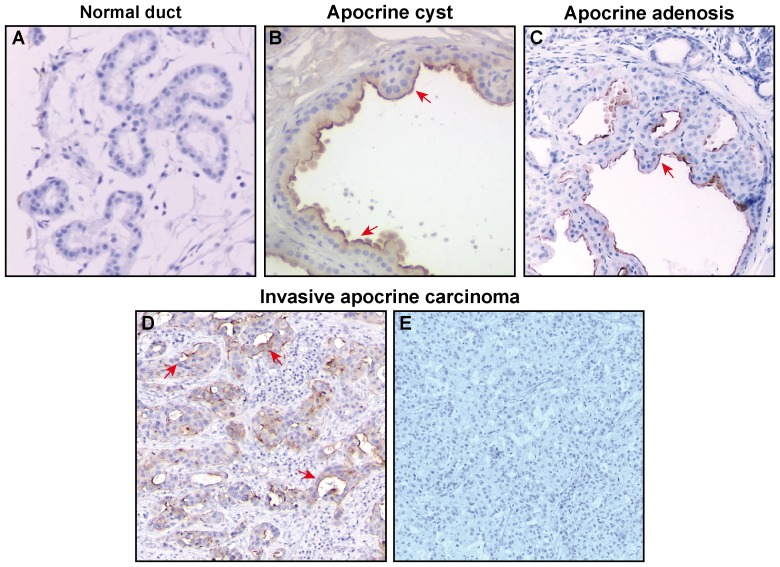
MLPH is expressed by non-malignant apocrine cells but is lost in IACs. Representative images of FFPE sections immunostained with antibody against MLPH. (A) normal ducts, (B) benign apocrine cysts (mainly luminal membranous immunoreactivity), (C) sclerosing adenosis with apocrine differentiation, (D) IAC showing positive immunostaining in pseudo-glands structures (cytoplasmic and luminal membranous immunoreactivity) and (E) IAC with negative immunostaining. Magnification: x20.

In order to identify whether or not these proteins are exclusively expressed in breast lesions with apocrine differentiation including IACs, we performed IHC analysis using tissue microarrays (Pantomics Inc.) of non-IAC breast tumor subtypes which we used in our previous experiments (see above). The results are summarized in Figure 8 and demonstrate that the protein expression of 8 genes from the transcriptomic signature, namely, FOXA1, XBP1, BLVRA, ABCA12, SIDT1, RHOB, SLC2A10, TSC22D3 is not apocrine-specific, because those proteins are frequently expressed in other types of breast cancer (Figure 8). Only 2 proteins, MLPH and SLC7A8, displayed low frequencies of positivity in non-apocrine breast tumor subtypes (2,9% and 9,7% respectively; Figure 8). To estimate the value of MLPH and SLC7A8 as putative markers for apocrine differentiation in breast, we screened their expression in the sets of breast normal tissue (the set is presented in Table S1), apocrine cysts (the set is presented in Table S2) and independent set IACs (17 samples) collected from the Department of Pathology, Rigshospitalet Copenhagen University Hospital. The representative IHC sections are shown in Figure 9. All normal ducts and IACs were negative for MLPH. However, 8 out of the 14 apocrine cysts (57%) showed positive immunoreactivity for MLPH with a pattern restricted to the membrane/cytoplasm of luminal cells in apical snouts (Figure 9B). This pattern was also observed in sclerosing adenosis with apocrine differentiation (Figure 9C). Interestingly, MLPH positive expression was still retained by pseudo-glandular structures present in one IAC sample (Figure 9D), suggesting that loss of expression of MLPH protein in IACs may be due to a process of dedifferentiation or loss of the glandular structure. As well, 87% of non-malignant samples and 63% apocrine cysts displayed negative staining with the antibody against SLC7A8. A very weak cytoplasmic staining was observed in 30% of cysts. Notably, all 17 IAC samples were negative for SCL7A8. It is not inconceivable that MLPH and SLC7A8 proteins are expressed and function in apocrine carcinoma at a very low level but, consequently, all oscillations in their expression are below the resolving power of the IHC.

## Discussion

The accurate diagnosis of breast apocrine carcinoma remains controversial, mainly due to the rather subjective histopathological criteria and the lack of sensitive and specific biomarkers, which can reliably categorize this subtype of breast carcinoma. The strategy we have employed to generate protein markers to specifically categorize IAC and potentially be used as targets for therapy, is based on the assumption that these lesions arise from apocrine cells, which in turn are derived from normal breast epithelial luminal cells that have undergone apocrine metaplasia [1], i.e. transition from breast epithelial cells into an apocrine sweat-gland type of cells [2], [19], [28]. Here we report an analysis of the expression of two novel putative protein biomarkers, FABP7 and HMGCS2, in breast lesions undergoing apocrine differentiation: from benign apocrine metaplasia to invasive apocrine carcinoma.

We have found that the lesions with apocrine metaplasia as well as apocrine cysts in breast were highly positive for both HMGCS2 and FABP7 as compared to normal non-apocrine mammary epithelial cells (p<0.001 and p<0.0001, respectively), implying their value as novel biomarkers for breast apocrine differentiation. HMGCS2 and FABP7 in combination with the panel published in our previous studies, 15-PGDH+, HMGCR+ and ACSM1+ [2], [16], [19], [28] may represent the golden standard for defining the breast apocrine phenotype. Expression of FABP7 and HMGCS2 by invasive apocrine cancer was further demonstrated by IHC using a well characterized set of apocrine carcinomas [2] in which more than 90% of the tumor cells exhibited cytological features typical of apocrine cells [21]. FABP7 positives were found in 78% of all IAC cases and in 96% of ADCIS and only in 14 out of the 210 non-IAC breast tumor subtypes (6,6%). Tang and coauthors [36] reported that FABP7 overexpression exhibited a strong relationship with triple-negative cases (45,8%, p = 0.001) and the basal-like subtype (37,5%, p = 0.001). Our results confirmed partially a higher frequency of FABP7 positives in TNBC group (∼19%) as compared to the other subtypes of breast tumors (Figure 6B). These findings are also in the line with the studies showing that FABP7 is associated with the basal phenotype and patient outcome in human breast cancer [37], [38]. Importantly, our data have demonstrated various sub-cellular localization of FABP7 in apocrine carcinomas and have shown that, unlike ADCIS, in which FABP7 was detected mainly in the cytoplasm, in IACs FABP7 was also observed in nuclei, which is in the line with recent data demonstrating that nuclear location is associated with a more aggressive phenotype of breast cancer [37], [38]. It was also shown, that FABP7 overexpression is correlated with pure glioblastoma histology and that nuclear expression of FABP7 is more specifically associated with more invasive tumors [39], [40]. Taken together these data support the contention that translocation of FABP7 between nuclei and cytoplasm may play a role in tumor progression and further investigation should be undertaken to understand roles of FABP7 signaling and intracellular traffic in tumor biology.

To our knowledge, there are no data available on the expression and activity on the protein level of HMGCS2, the gene that controls the anabolic ketogenic pathway in breast cancer. Here we have shown for the first time that HMGCS2 was up-regulated in ADCIS and IAC and only rarely found in non-apocrine breast carcinoma (Figure 6). Notably, HMGCS2 expression is retained by all the IACs from our morphologically-defined cohort. This stresses the potential value of HMGCS2 as a breast apocrine cancer biomarker, since other apocrine differentiation markers such as 15-PGDH, ACSM1 and HMGCR reported by us previously, are expressed consistently in benign apocrine lesions but are conserved at lower frequencies or even negative in the invasive stages [19]. Recently, Wang and co-authors using Illumina expression array/RT PCR analysis sought to identify risk biomarkers that are specific to ER status of breast cancer and among several others revealed a significant overexpression of HMGCS2 in ER- cases [41]. However, our data did not confirm overexpression of HMGCS2 in ER negative tumors at the protein level: the group of TNBCs displayed only ∼2% positives for HMGCS2 (Table S4).

IAC as defined by morphological criteria is often characterized by a specific hormone receptor signature: AR+/ER-/PR- [9], [42], [43], [44], [45] that has been proposed as a marker for apocrine–type tumors by Tsutsumi and coauthors, who suggested to include androgen receptor to immunohistochemical criteria [46]. However, several studies reported a significant proportion of AR+/ER-/PR- phenotypes outside breast apocrine malignancy: 59% in HER2+ type and 32% in basal-like tumors [31]; 39% in ER- [47]; 10% in TNBC [10]. Our IHC data revealed only 22 AR+ER-PR- phenotypes out of the 205 non-apocrine samples (10.7%) present almost exclusively in TNBC patients (6) and HER2+ (14) samples. These discrepancies could be in part due to the lack of consistent application of the morphological criteria and the use of different methods and cut-offs values for positivity [9] suggesting that hormonal status AR+/ER-/PR- may not be sufficient to discriminate IAC from other breast cancer subtypes. The results presented here demonstrate for the first time a very high frequency of HMGCS2+/AR+/ER-/PR- phenotype (78,8%) in IAC diagnosed by classical morphological criteria as compared with the non-IAC control set (3%). We suggest that this four-protein signature can categorize breast apocrine carcinoma more confidently and supports the contention of a strict definition of pure apocrine carcinomas as it was suggested previously [48].

The identification of differentially expressed proteins that characterize the progression from early benign apocrine lesions to invasive stages opens a window of opportunity for designing and testing new approaches for pharmacological intervention as, for example, 15-hydroxyprostaglandin dehydrogenase (15-PGDH) and 3-hydroxymethylglutaryl-CoA reductase (HMGCR) are currently being targeted for chemoprevention strategies in various malignancies [49], [50], [51]. HMGCS2 is one of the rate-limiting enzymes controlling generation and re-utilization of ketone bodies [52]. Recently it was shown that ketone bodies support the driving of neoplastic growth which is often accompanied by starvation of all components of tumor environment and it has been speculated that ketone inhibitors can be designed as novel therapeutics to effectively treat advanced cancer patients [53], [54]. Consequently, HMGCS2, the enzymes associated with ketone body production and re-utilization, might be considered as new “druggable” targets for anticancer therapy. Our finding that IACs are characterized by an overexpression of HMGCS2, suggests that the patients diagnosed with apocrine carcinoma might benefit from therapy with HMGCS2 inhibitors. In addition, the high levels of AR expression in the IACs makes this receptor a promising anticancer therapeutic target for this type of breast cancer, but the ability to exploit AR for therapy remains to be challenging [55], [56], [57], [58]. Given that AR and HMGCS2 are both overexpressed in IAC, the use of the dual therapy targeting two parallel AR and HMGCS2 pathways may provide an additional benefit for therapeutic attack of breast apocrine carcinoma. HMGCS2 is the only gene from the breast apocrine protein marker panel identified in our studies that has been included in the molecular apocrine transcriptomic signature [14], and was also found among the genes which transcription was affected by modulation of AR/FOXA1 axis in apocrine cells [59]. It is plausible, based on these findings, to consider this enzyme as a "marker" to indicate an active AR, which can be used to predict which tumors are driven by this receptor and, therefore, capable of being treated with anti-androgen therapy.

To date, there is uncertainty regarding the relationship between molecular apocrine tumors identified by mRNA-array studies [12], [13], [14] and histopatologically–diagnosed apocrine malignancies. Thus, Lehman-Che and co-authors found only 4 tumors that meet morphological criteria for apocrine tumors among 58 patients diagnosed as “molecular apocrine” [15], suggesting that molecular apocrine subtype is much broader than initially reported by Farmer and co-authors [12]. Apocrine transcriptomic signatures contain several genes related to signaling pathways (RHOB and SIDT1), transcription factors (FOXA1, XBP1, TSC22D3) and transporters (ABCA12, SLC2A10 and SCL7A8) that are not observed in our proteomic signature and therefore were of interest to study. Our reciprocal IHC analysis of a set of 10 proteins selected from the major top candidates of molecular apocrine signature: FOXA1, XBP1, BLVRA, ABCA12, SIDT1, MLPH, RHOB, TSC22D3, SLC2A10 and SLC7A8 has revealed only one protein, melanopholin (MLPH), an adapter protein involved in melanosome transport to cell periphery [60], which expression was specific for benign apocrine lesions, but was lost in invasive stages. Most of other selected candidates were expressed on protein level at different frequencies among non-IAC samples, presumably because their function is related to housekeeping tasks. The differences in concentration of mRNAs and proteins within a cell is on average from 17 to over 50,000 copies, respectively [61], and most studies agree that mRNA levels can explain only about 30–60% of protein abundance, so regulation of post-transcription, translation and protein processing contribute as much to variation in protein concentrations as transcription and transcript degradation do [62]. On the contrary, as we have seen for HMGCS2 and FABP7, and previously for 15-PGDH, HMGCR, ACSM1 and other proteins [2], [16], the secondary metabolism of lipids, amino acids and carbohydrates is strongly represented in apocrine cells, providing a clear cut differentiation from other cell types of the breast. Therefore, the unique metabolic identity of the apocrine cells has offered so far the best candidates to identify IAC biomarkers.

In conclusion, HMGCS2 and FABP7 are novel protein markers for apocrine differentiation that are highly conserved among apocrine breast tumors retaining their expression during tumor progression. Inclusion of these markers into the protein signature described previously: 15-PGDH, ACSM1 as well as markers that are consistently expressed (AR, CD24) or not present (ER alpha, PR, Bcl-2, and GATA-3) in benign apocrine lesions [19], [28] may categorize ADCISs and IACs with far more accuracy. Combination of HMGCS2 with steroidal profile (AR+/ER-/PR-) offers greater sensitivity to detect breast apocrine tumors compared to steroidal profile alone. Moreover, HMGCS2 widespread expression among IACs makes it a candidate to be studied as potential therapeutic target for this malignancy. Integration of proteomic and transcriptomic technologies applied to apocrine breast cancers may offer complementary information about the particular biology of this tumor subtype and the identification of biomarkers and therapeutic targets of clinical importance.

## Materials and Methods

### Sample collection and handling

Tissue samples from 412 high risk breast cancer patients that underwent breast surgery were obtained from the Department of Pathology, Rigshospitalet, Copenhagen University Hospital between 2003 and 2014. The criteria for high risk cancer applied by the Danish Cooperative Breast Cancer Group (www.dbcg.dk) are age below 35 years old, and/or tumor diameter of more than 20 mm, and/or histological malignancy grade 2 or 3, and/or negative estrogen and progesterone receptor status, and/or positive axillary status. All patients did not receive preoperative treatment. The samples were routinely collected within about 30–45 min from the time of surgical excision. To evaluate the content of tumor cells and the ration of tumor/stroma the hematoxylin/eosin stained slides were prepared and reviewed by two researches (IIG and M-L T). The age range was 32–84 with a mean average of 59 years. All 412 samples collected during the above period were subjected to 2D PAGE analysis. The samples were placed in dry ice and were rapidly transported to the Danish Cancer Society Research Center where they were stored at −80°C. Apocrine carcinoma was morphologically defined as a carcinoma in which more than 90% of the tumor cells showed typical apocrine features. TNBC, Luminal B and HER2+ tumor subtypes were defined in accordance with St. Galen classification [63]. The project was approved (KF 01-069/03) by the Copenhagen and Frederiksberg regional division of the Danish National Committee on Biomedical Research Ethics. The written informed consent from the donor was obtained for the use of the samples in research. In addition, one set of specific samples comprising 14 ADCISs and 33 IACs diagnosed between 1997 and 2001 used in this study have been described in previous publications [21], [29]. Breast cancer tissue microarray slides were obtained from Pantomics (BRC1501, BRC1502 and BRC1503 Pantomics Inc., CA, USA). The TMAs contain of 210 non overlapping breast tumors in total.

### Antibodies

The polyclonal antibody raised against synthetic peptides of human FABP7 (HPA028825), BLVRA (HPA042865), ABCA12 (HPA043194), SIDT1 (HPA035862), MLPH (HPA014685) were purchased from Atlas Antibodies and were used in IHC at a dilution of 1∶200, 1∶100, 1∶500, 1∶50 and 1∶100 respectively in accordance to the manufacturer instructions. The polyclonal antibody against HMGCS2 (XW-7255) was obtained from BioSite (Sweden) and was used in IHC at a dilution of 1∶500. The polyclonal antibody raised against synthetic peptides of human FOXA1 (ab23738), XBP1 (ab37152) and RHOB (ab75064) were purchased from Abcam and were used in IHC at a dilution of 1∶400, 1∶300 and 1∶2000 respectively. The polyclonal antibody against SLC2A10 (LS-B1622) and monoclonal antibodies against TSC22D3 (LS-B4313) were purchased from LSBio and were used in IHC at a dilution 1∶2000 and 1∶2000 respectively. The polyclonal antibody raised against synthetic peptide of human SLC7A8 (BMP041) was obtained from MBL and was used in IHC at a dilution 1∶500. All dilutions were used according to the manufacturer's instruction. The antibodies against 15-PGDH, ACSM1, GCDRP15, ER, PR, AR, HER2, 15-PGDH, ASCM1, CD24, Bcl2 and GATA3 are described in our previous studies [19], [28].

### 2D PAGE and 2D Western immunoblotting

Twenty to thirty, 6 µm cryostat sections of frozen tissues were resuspended in 0.1 ml lysis solution [64] or CLB1 buffer [65]. The resulting lysates were frozen and kept at −20°C until used [66]. Twenty to forty µl were applied to the gels and each sample was run at least in duplicate. The first and last sections of each sample were used for IHC analysis using cytokeratin 19 (CK19) antibodies to trace possible changes in epithelial cell pattern during tissue sectioning. (CK19 is ubiquitously expressed by mammary epithelial cells [67], [68] and can serve as an additional control for the sample quality. 2D PAGE (isoelectric focusing, IEF), silver staining and 2D gel Western blotting were performed as previously described [69].

### Image analysis

For comparative proteomic analysis 2D gels were scanned and analyzed using the PDQUEST software package from BioRad (version 8.0.1). All detected proteins were identified by mass spectrometry (see below), selected for the comparative analysis and added to the master image.

### Mass spectrometry analysis

Protein spots were excised from silver stained dry gels and the gel pieces were re-hydrated in water. Gel pieces were detached from the cellophane film and cut into 1 mm^2^ pieces followed by “in-gel” digestion as previously described [70] followed by a procedure that has been reported previously [71]. Briefly, MALDI-TOF-TOF data were acquired using an Ultraflex III 200 time-of-flight mass spectrometer (Bruker Daltonik, Germany) equipped with a Smart beam laser and a LIFT-TOF/TOF unit. Data acquisition and data processing were performed by the Flex Control 3.0 and Flex Analysis 3.0 software (Bruker Daltonik, Germany). All of the spectra were obtained using reflector positive mode with an acceleration voltage of 25 kV, reflector voltage of 26.38 kV and detection suppressed up to 450 Da. A total of 2000 shots in steps of 200 shots were added to one spectrum in the mass range of *m/z* 600–4000. Spectral analysis and protein identification were performed as described previously. Protein identifications were considered to be confident when the protein score of the hit exceeded the threshold significance score of 65 (p<0.05) and no less than 6 peptides were recognized.

### Immunohistochemistry

The procedures for IHC have been described in detail in previous publications [2], [21], [29]. For FABP7 and HMGCS2 we used the following cut-off values: ADCIS: positive if 60% or more of the CIS contained at least 30% of the cells reacting with the antibodies. IACs: positive if 30% or more of the invasive cells reacted with the antibodies. The cut-off values for ER, PR, AR, HER2, 15-PGDH, ASCM1, CD24, Bcl2 and GATA3 are used as described previously [19]. For XBP1, TSC22D3, ABCA12, SIDT1, FOXA1, RHOB, BLVRA, SLC2A10, SLC7A8 and MLPH we used the following cut-off values: positive if 10% or more of the cells showed a clear positive staining with the antibodies. TMAs were analysed by scoring of up to 5 separate areas per single core and the intensities were averaged to generate a summary score (percentage of cells stained, intensity). Frequency distribution analyses were performed on GraphPad Prism 5 software (GraphPad Software, Inc, San Diego, CA, USA) using Fisher's exact test, considering a P value <0.05 as statistically significant. Immunohistochemical profiles were constructed using Multiexperiment Viewer MeV [72].

### Immunofluorescence on paraffin sections

Five-µm sections cut from paraffin blocks of breast tissue samples were mounted on Super Frost Plus slides (Menzel-Gläser, Braunschweig, Germany), baked at 60°C for 60 min, deparaffinized, and rehydrated through graded alcohol rinses. Heat induced antigen retrieval was performed by immersing the slides in Tris/EDTA pH 9.0 buffer (10 mM Tris, 1 mM EDTA) and microwaving in a 750 W microwave oven for 8 min. Following antigen retrieval sections were treated with Image-iT FX signal enhancer (Molecular Probes, USA) to block non-specific staining and subsequently incubated with the relevant primary antibodies at appropriate dilutions. Detection of immune complexes was done with species specific secondary antibodies conjugated to Alexa Fluor 488 and Alexa Fluor 594 (Molecular Probes, OR, USA). Nuclear material was counterstained with TO-PRO-3. The sections were washed three times with cold phosphate-buffered saline (PBS) between incubations. Normal rabbit or mouse serum instead of primary antibody was used as a negative control. Sections were imaged using a laser scanning microscope (Zeiss LSM510Meta) as previously described [73].

### IHC blocking with recombinant FABP7 and HMGCS2

The human recombinant FABP7 (H00002173) and HMGCS2 (H00003158) proteins were purchased from Abnova (USA). The appropriate amount of corresponding antibodies (see above) were diluted in TBS buffer to the final volume needed for staining of two section slides and divided equally. The blocking recombinant proteins FABP7 and HMGCS2 were added to a final concentration 5 µg/ml providing approximately 5-fold molar excess of blocking peptide. Tubes containing either pure antibody or the antibody mixed with corresponding recombinant protein were incubated at room temperature for 1 hr with agitation. Two identical tissue sections were probed with the blocked antibody and antibody alone as described in the Immunohistochemistry section.

### Breast cancer cell lines

Human breast cancer cell line MDA-MB-453 was obtained from the American Type Culture Collection (ATCC, Manassas, VA, USA). Cells were cultured in Dulbecco's modified Eagle's medium (DMEM, Gibco-BRL, Invitrogen, Carlsbad, CA, USA) supplemented with 10% fetal bovine serum (FBS) at 37°C in a humidifed CO_2_ incubator. The cell pellet from exponentially growing breast cancer cells was collected by centrifugation at 1000 g to prepare the FFPE block. IHC staining was performed as described above.

## Supporting Information

Figure S1 **Validation of anti-FABP7 antibodies specificity towards highly homologous members of human fatty acid-binding protein family.** (A) 2D silver stained gel of proteins extracted from tumor tissue obtained from TNBC patient 22. (B) Zoomed fraction of area shown in (A) by dash rectangle. Four members of FABP family, namely, FABP3, FABP4, FABP5 and FABP7 are indicated by red. Several other neighboring proteins are indicated as references. The identity of all proteins was determined by mass spectrometry analysis. (C) 2D Western blot of 2D gel shown in (A). Only the fraction of blot image corresponding to the area shown in (B) is presented. The antibodies recognize only FABP7 but do not cross react with FABP3, FABP4 and FABP5.
(TIF)

Figure S2 **Immunohistochemical expression analysis of FABP7 and HMGCS2 in MDA-MB-453 cell line.** The FFPE sections from breast cancer cell line MDA-MB-453 were immunostained with antibodies against FABP7, HMGCS2, 15-PGDH, ACSM1 and GCDFP-15. Magnification: x20.
(TIF)

Table S1 Expression of FABP7 and HMGCS2 in 28 normal breast lesions adjacient to tumor.
(XLS)

Table S2 Expression of FABP7 and HMGCS2 in 13 breast apocrine cysts.
(XLS)

Table S3 Expression of FABP7, HMGCS2 and several other markers by 14 ADCISs.
(XLS)

Table S4 Expression of FABP7, HMGCS2 and several other markers by 33 IACs.
(XLS)

Table S5 Expression of FABP7, HMGCS 2 and several other markers in various nonapocrine breast tumors as determined by IHC of TMA array (Pantomics 1501, 1502 and 1503).
(XLS)
