# Supplementary figures and images for: FABP7 and HMGCS2 Are Novel Protein Markers for Apocrine Differentiation Categorizing Apocrine Carcinoma of the Breast
Source: PLoS One. 2014 Nov 12;9(11):e112024. doi: 10.1371/journal.pone.0112024 (PMC4229141; doi:10.1371/journal.pone.0112024)

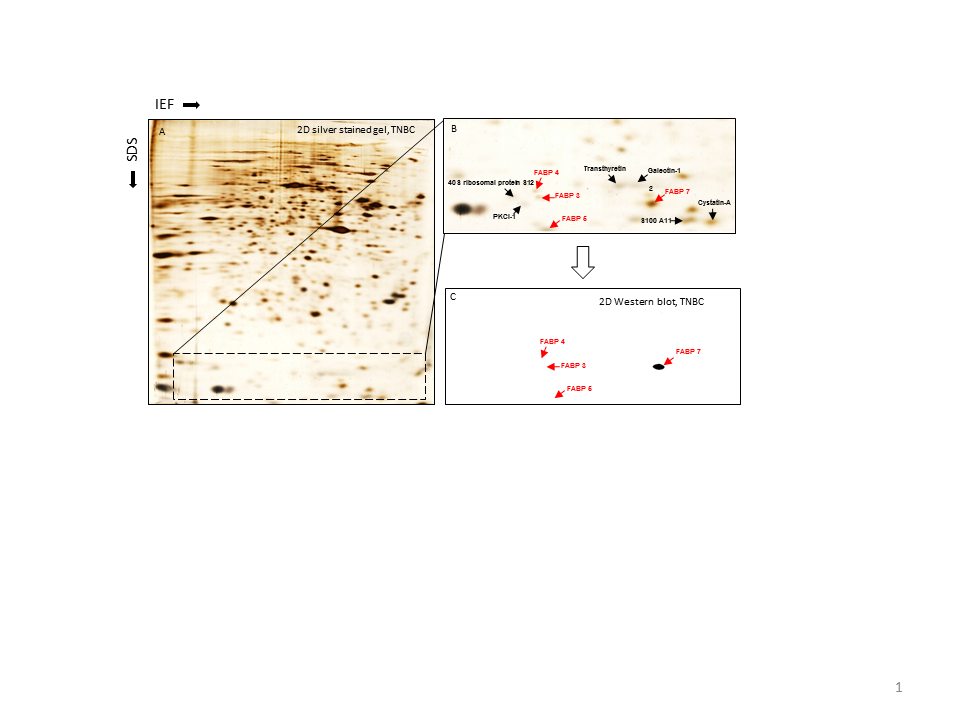

Supplement: Figure S1 — Validation of anti-FABP7 antibodies specificity towards highly homologous members of human fatty acid-binding protein family. (A) 2D silver stained gel of proteins extracted from tumor tissue obtained from TNBC patient 22. (B) Zoomed fraction of area shown in (A) by dash rectangle. Four members of FABP family, namely, FABP3, FABP4, FABP5 and FABP7 are indicated by red. Several other neighboring proteins are indicated as references. The identity of all proteins was determined by mass spectrometry analysis. (C) 2D Western blot of 2D gel shown in (A). Only the fraction of blot image corresponding to the area shown in (B) is presented. The antibodies recognize only FABP7 but do not cross react with FABP3, FABP4 and FABP5. (TIF) [file pone.0112024.s001.tif]

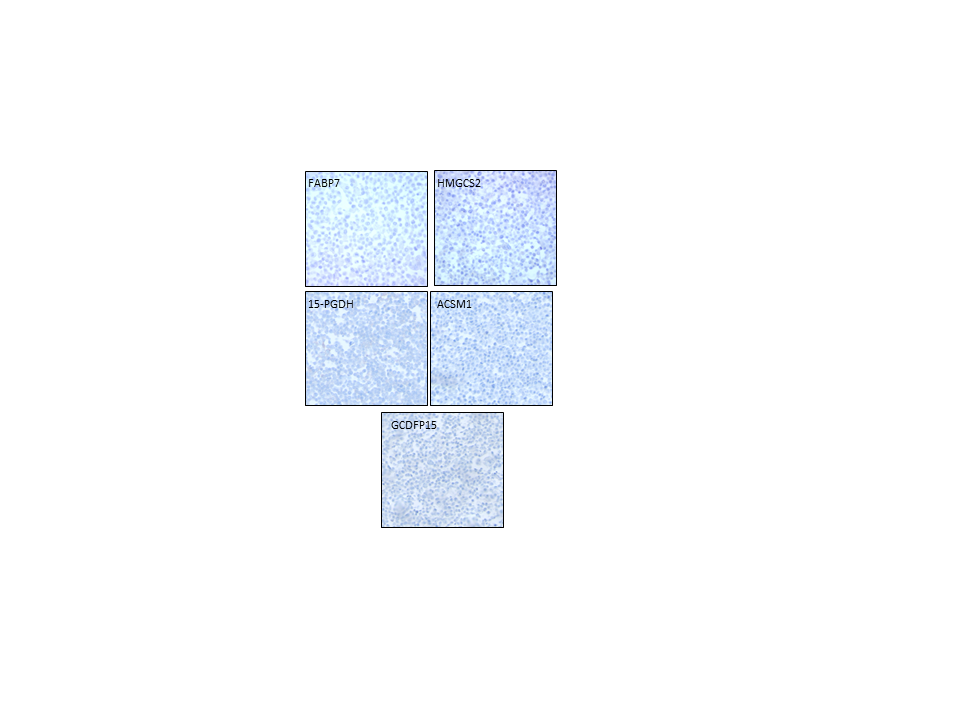

Supplement: Figure S2 — Immunohistochemical expression analysis of FABP7 and HMGCS2 in MDA-MB-453 cell line. The FFPE sections from breast cancer cell line MDA-MB-453 were immunostained with antibodies against FABP7, HMGCS2, 15-PGDH, ACSM1 and GCDFP-15. Magnification: x20. (TIF) [file pone.0112024.s002.tif]
